# Return to sport after patellar dislocation or following surgery for patellofemoral instability

**DOI:** 10.1007/s00167-014-3172-5

**Published:** 2014-07-22

**Authors:** Jacques Ménétrey, Sophie Putman, Suzanne Gard

**Affiliations:** 1 Centre de medicine de l’appareil locomoteur et du sport, Unité d’Orthopédie et Traumatologie du Sport (UOTS), Swiss Olympic Medical Center, Service de chirurgie orthopédique et traumatologie de l’appareil moteur, University Hospital of Geneva (HUG) and Faculty of Medicine, University of Geneva, Rue Gabrielle-Perret-Gentil 4, 1211 Geneva, Switzerland; 2 Université Lille Nord-de-France, 59000 Lille, France; 3 Département universitaire de chirurgie orthopédique et de traumatologie, Hôpital Salengro, CHRU de Lille, rue Émile-Laine, 59037 Lille, France

**Keywords:** Patellofemoral, Return to play, Rehabilitation, Dislocation, Dynamic stability

## Abstract

Patellofemoral instability may occur in a young population as a result of injury during sporting activities. This review focuses on return to sport after one episode of dislocation treated no operatively and as well after surgery for chronic patellofemoral instability. With or without surgery, only two-thirds of patients return to sports at the same level as prior to injury. A high-quality rehabilitation programme using specific exercises is the key for a safe return to sporting activities. To achieve this goal, recovery of muscle strength and dynamic stability of the lower limbs is crucial. The focus should be directed to strengthen the quadriceps muscle and pelvic stabilizers, as well as lateral trunk muscle training. Patient education and regularly performed home exercises are other key factors that can lead to a successful return to sports. The criteria for a safe return to sports include the absence of pain, no effusion, a complete range of motion, almost symmetrical strength, and excellent dynamic stability.

*Level of evidence* IV.

## Introduction

Patellar dislocation and patellofemoral instability may occur in a young population with varying activity levels. The overall recurrence rate of patellar dislocation after an initial event is close to 40 % [23]. In fact, patients who have a primary patellar dislocation have a 17 % recurrence rate, and patients who sustain repeat patellofemoral joint dislocation have a 49 % recurrence rate [13]. Therefore, surgical treatment is generally recommended after a second dislocation [14]. It is obvious that athletic patients have as a goal resumption of their sports activities at the same high level as prior to the episode of dislocation. For less active patients, an increase in their level of activity could be an attractive goal, as strength and stability of the lower limb can prevent re-injury [18]. Information about the functional capacity that allows for a safe return to sports after either primary dislocation or surgical stabilization is sparse. It is known that medial patellofemoral ligament (MPFL) reconstruction and rehabilitation improves the ability to perform routine activities of daily living [8, 13], but it is less evident as regards the ability to resume sports participation safely. Testing protocols are requested after patellar dislocation to assure a safe return to sport (RTS), but information on this issue is currently limited in the literature. Thus far, quantitative assessment was recommended, but as it will be shown in this article, qualitative measurement with a systematic video analysis has to be considered to better evaluate the dynamic stability of the knee.

This article focuses on objective patellofemoral instability and return to sport, addressing only those situations where one or more episodes of dislocation have occurred or in cases where surgery for instability has been performed. It was attempted to answer the following two questions: (1) How and when can patients safely return to sport after primary or recurrent patellar dislocation, or after patellofemoral stabilization? (2) What validated evaluations can be utilized to determine readiness to return to sport? In our experience, return to sport can be compromised by pain, instability, weakness, and poor motor control.

## Return to sport after nonsurgical treatment

A safe return to sport implies that lesions of the knee have healed and the injured lower limb has adequately recovered to face the demands of sporting activities. Successful return to sport implies: (1) no early re-injury; (2) no further damage to the knee; (3) return to the preinjury or higher level; (4) no limiting pain; (5) still playing after 5 years; and (6) no early osteoarthrosis. A comprehensive return to sport decision-making process should be based upon: (1) clinical examination; (2) evaluation of laxity; (3) strength measurement; (4) neuromuscular evaluation; and (5) counselling with the physical conditioner and coach for professional athletes. As regards the patellofemoral joint, the challenge is that the patella is a sesamoid bone enclosed within the extensor mechanism of the lower limb. Its function is closely associated with dynamic muscle activity while retaining its osseous and soft tissue static elements [26].

Atkin et al. [3] followed 74 patients (37 men, 37 women) after a first dislocation that was treated conservatively. Preinjury sports activity was similar to that of patients with primary anterior cruciate ligament (ACL) injury [10]. Patients were authorized to return to sports after they regained full passive range of motion, had no effusion, and when quadriceps muscle strength was at least 80 % of the noninjured limb. Patients regained range of motion after 6 weeks. Sports participation was limited during the first 6 months after injury, with difficulties in squatting and kneeling. At 6 months, 58 % of patients noted limitations in strenuous activity, but no recurrence was recorded. Sillampaa et al. [32] reported that the return to preinjury activity level varied between 44 % and 60 % regardless of the modality of treatment after the first dislocation.

### Critical points in the rehabilitation for achieving a proper return to sport

There are few high-quality studies concerning rehabilitation after patellar dislocation [33], especially as regards the last phase of treatment before return to sport. There are no published randomized clinical trials, but only thoughts and recommendations as summarized in the review by Fisher et al. [13]. However, since the objectives are similar, a parallel situation exists with the information available in the literature concerning return to sport after ACL reconstruction. In order to achieve a proper and safe resumption of sports activities, there are critical points to achieve during rehabilitation. The strength of the lower limb muscles, especially the quadriceps [6, 36], and the gluteus medius is one key point [5]. Core strength is also crucial as it plays an important role in the stability of the lower limb. Indeed, if the trunk is not stable during cutting manoeuvres, the loads applied to the knee are in valgus, thus generating a situation where the patellofemoral joint is at risk of dislocation [13, 18].

To avoid re-injury, the stability of the lower limb must be mastered at the end of the rehabilitation programme. Cutting manoeuvres, change of direction, and running on uneven ground are the three activities perceived to be the greatest risk factors for patellar dislocation [34]. Therefore, the final goal of the rehabilitation programme should be to focus on the stability of the lower limb by the use of specific exercises on different surfaces, including cutting manoeuvres, side hops, and sudden change of direction.

During the final phase of the rehabilitation programme, another important factor to consider for a proper return to sport concerns sport-specific activities. While this may seem obvious, it is often overlooked. The athlete should be prepared for the specific loads and demands to be experienced in their specific sport, including: (1) cutting manoeuvres and pivoting exercises, performed for most of the team sports; (2) plyometric and landing strategies, emphasized in any sports with jumps; (3) one-leg stability, particularly exercised for martial arts; and (4) proprioception, side stability, and landing capacities, which are stressed with skiers. As previously mentioned, a parallel can be made with return to sport after ACL reconstruction. Specific exercises to be implemented for patients involved in soccer, basketball, alpine skiing, and American football have been described [7, 20, 37, 38].

## Return to sports after surgery for chronic patellofemoral instability

The same principles as mentioned above can be applied to patients after surgery for instability, regardless of the procedure performed. With respect to return to sport after medial patellofemoral ligament (MPFL) reconstruction, Fisher et al. [13] reviewed postoperative rehabilitation and the incidence of return to sport. They reported on only two studies [11, 25] concerning the rate of return to a specific level of sports and found that 77 % of patients were able to resume sports at their preinjury level of performance. In a study by Ntagiopoulos et al. [29], 87 % of patients returned to their previous activities after isolated trochleoplasty, but the level of activity was not specified. Nelitz et al. [27] studied the outcome after combined trochleoplasty and MPFL reconstruction for recurrent dislocation in patients with severe trochlear dysplasia. Twenty-eight patients at a minimum follow-up of 2-years were included in the study. One patient returned to sport at a higher level than preoperatively, sixteen returned at the same preoperative level, and only six patients reported a return to a lower level of activity than before surgery. Overall, 60 % of patients returned to their previous activities. Unfortunately, there is no information in the literature about validated timelines for a safe return to sport after patellofemoral surgery, especially after tibial tubercle osteotomy. However, common sense recommends to wait until the osteotomy is healed, and to delay the return until maximum recovery of muscle strength and dynamic stability.

Cartilage lesions represent a critical factor when functional recovery is considered. Treatment can vary according to the size of the lesion and the intrinsic stability of the patellofemoral joint [12]. Factors to consider include size of the lesion, intrinsic healing capacity of the cartilage, and residual pain. Cartilage lesions are certainly not a favourable factor for a safe return to high-impact and high-demanding sports activities. Clearly, a large cartilage lesion (>2 cm^2^) influences the prognosis for return to sport. However, there is no specific data in the literature that allows for solid recommendations.

Based on the current literature, the return to previous level of sports activities after an objective episode of instability is limited to two-thirds of patients with or without surgery.

## Discussion

### Criteria for a safe return to sport

There is little in the current literature about return to sport after objective patellar instability, and thus, no clear criteria can be as yet endorsed. However, as already mentioned, one can draw a parallel with the literature dedicated to return to sport post-ACL reconstruction [7, 20, 37, 38]. Based upon this literature and our experience, we propose six clinical criteria to support our return to sport decision-making process. As regards timing, it is no longer a matter of weeks or months, but rather a matter of clinical and testing requirements that the patient should fulfil. These criteria are as follows: (1) no pain; (2) no effusion; (3) no patellofemoral instability; (4) a full range of motion; (5) nearly symmetrical strength (85–90 %); and (6) excellent dynamic stability. These criteria can be applied in patients treated with or without surgery. Ideally, patients should satisfy these criteria at 6 weeks after a dislocation, and 3 months after surgery. Our protocol and criteria for return to sport are currently under evaluation. In 2013, the ISAKOS Sports Medicine Committee also defined criteria for return to sport after surgery, and their criteria are very similar to ours (Table 1). Initially, the first 4 criteria listed above are assessed by the surgeon, followed by measurement of quadriceps and hamstring muscle strength in both legs with an isokinetic dynamometer. At this point, dynamic stability is assessed with the use of several functional tests and video recording. These tests typically use the patient’s contralateral limb as the control or “normal”, and a limb symmetry index (LSI) is thus calculated by computing side-to-side differences in the results. And finally, in light of the results, the surgeon and patient can make a well-documented decision with regards to return to sport. For strength evaluation, especially in high-demanding sports (e.g., alpine skiing, football, basketball, and handball), the patient should reach a LSI of at least 90 % in order to be cleared for return to sports [7, 20, 37, 38].

**Table 1:** From the ISAKOS Sports Medicine Committee, ISAKOS Consensus Meeting on Return-to-Play, London 2013. With the permission of Elisabeth Arendt, MD and François Kelberine, MD

Criteria for a safe RTS after patellofemoral instability ISAKOS
If bony surgery is involved, complete radiographic healing of bone
No complaints of knee pain or knee instability
Full or near full range of motion
No knee effusion
Completed neuromuscular training/proprioception
Satisfactory core strength and endurance
Acceptable control with dynamic activities (e.g., Star Excursion Balance Test)
Limb Symmetry Index > 85 % on hop tests, especially if resuming pivoting sports
Adequate performance with physiotherapist during sport-specific drills simulating the intensity and movement patterns of the athlete’s given sport
Athlete demonstrates a psychological readiness to return to sport (e.g., SANE score > 80/100)

Testing exercises are mandatory to evaluate recovery and the competence of the injured or operated limb. There are many functional tests for the knee that have been used [17, 22, 31, 39] and appear to be reliable and valuable tools [40]. After a thorough evaluation of the different tests that have been validated in the literature, we have selected the “single-leg squat”, the “Star Excursion Balance Test” (SEBT), the “drop jump test”, and the “side-hop test”. These tests are always performed after isokinetic measurements of the strength in both limbs.

**Fig. 1:**
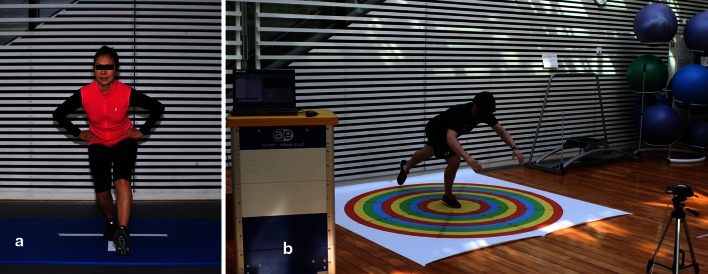
**a** Single-leg squat to evaluate the dynamic stability of the lower limb, **b** Star Excursion Balance Test(SEBT) to evaluate the limb stability while reaching the maximum distance around with the other leg

Dynamic stability can be evaluated with the single-leg squat (Fig. 1a) and the SEBT (Fig. 1b). In the single-leg squat, the knee must stay above the foot without going into a valgus movement, and the pelvis has to remain stable without dropping or turning. This exercise is part of the rehabilitation programme and should be perfectly mastered [24]. In the SEBT, the knee has to stay aligned as the pelvis is moving [35]. The patient stands on one foot and reaches points around him like a clock quadrant [18, 28]. The results obtained are qualitative on the video analysis and also quantitative since distances reached during the SEBT can be noted and reported. This test is very useful for the evaluation of lower limb stability [9, 16, 21].

**Fig. 2:**
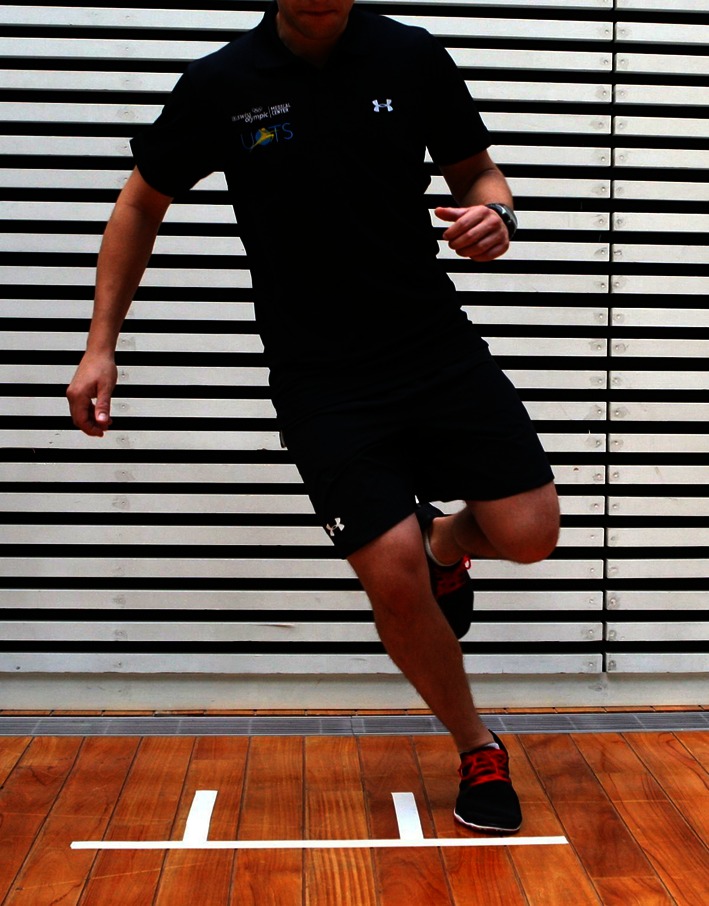
Side hop-test on lines 40cm away during 30 seconds. The trunk and limb alignement is observed

Many sports demand change in direction and landing from jumps. If the limb cannot be maintained in good alignment and as well properly decelerate, this may result in pain and risk re-injury [30]. It is therefore important to assess these parameters during the phase of return to sport. Drop and jump is a simple test that provides valuable information about control of landing. The patient drops from a box 35 cm high, lands on both feet, and immediately jumps as high as possible before landing a second time. The symmetry of the reception, the alignment of both knees, deceleration, and the capacity for absorbing the shock are evaluated using video recording [4, 15, 19, 30]. In this case, the results are qualitative, but highly valuable. The side-hop test consists in jumping on one leg between two lines at a distance of 40 cm as often as possible in 30 s (Fig. 2). During this test, many aspects of physical conditioning can be observed, including speed, agility, muscle coordination, limb alignment, trunk stability, and control in change of direction. Here, the results are qualitative (video analysis) as well as quantitative (number of jumps). All these tests are recorded and analysed using video analysis software. They are then shown to the patient, and the results discussed in order to outline a new set of exercises aimed at correction of any deficits noted. In this way, the training may evolve over time towards more sport-specific exercises with the aim to prepare the patient for return to sport. Arendt et al. have also described a series of tests that can be used to evaluate patients with patellofemoral problems or following corrective patellofemoral surgery [1, 2, 26]. They assessed core and trunk stability using prone plank, side plank, and single-limb exercises. For patients with patellofemoral problems, lower limb physical performance was evaluated with the stand and reach balance test, SEBT, single-limb squat test, and retro step-up/down test.

**Fig. 3:**
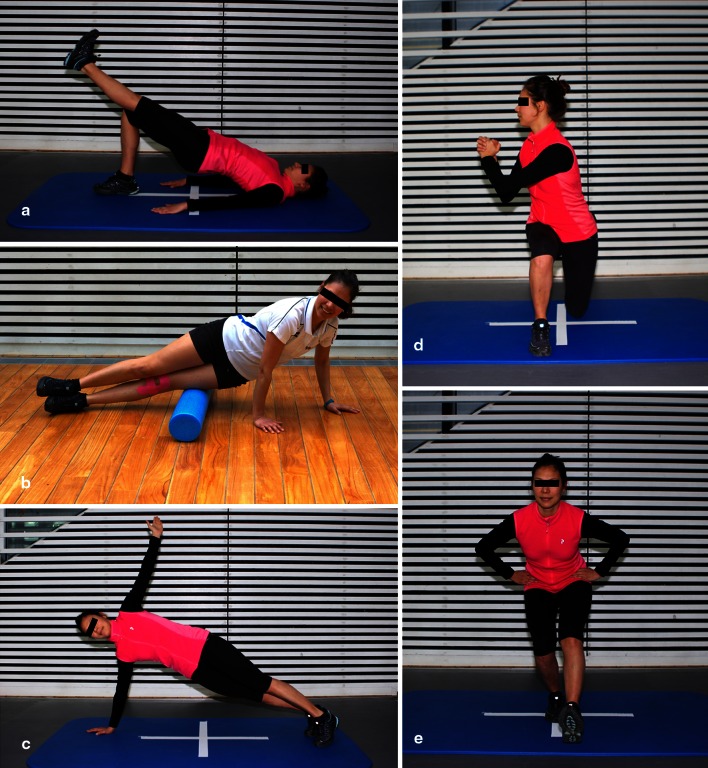
Home exercise programme: **a** Gluteus and hamstrings strengthening, **b** Myofascial release of ITB, **c** Rotational core stability, **d** Lunges with trunk rotation, **e** Single-leg squat

From our point of view, patient education is a key point. Very often, the patient has no idea about the importance of whole limb stability and is overly focused on the patellar problem. The use of video feedback is a very powerful tool for education. It is strongly recommended that patients regularly perform home exercises (Fig. 3a–e). Experience has shown that these self-administered exercises have been very useful to return the patient to sports.

With all, we have presented there still remains a lack of solid evidence about the criteria to determine a safe return to sports after patellofemoral dislocation or surgery. Further work needs to be conducted in this field to better understand the true capability of our functional testing, rehabilitation programme, and surgical procedures, to safely and durably bring our patient back to sports.

## Conclusion

Patellar instability is a pathology that concerns a young active population. Even after a single dislocation, return to sports at the same level of performance as before injury appears to be compromised. Analysis of the literature reveals that only two-thirds of patients return to sports at the same level. This can be considered a poor result with respect to the young age of the affected population. Return to sport may be encouraged and promoted using home self-administered exercises and by educating the patient about the importance of regaining muscle strength and dynamic stability. Finally, testing protocols for the RTS should include quantitative and qualitative criteria.
